# Dimeric chlorite dismutase from the nitrogen‐fixing cyanobacterium *C* *yanothece* sp. PCC7425

**DOI:** 10.1111/mmi.12989

**Published:** 2015-04-06

**Authors:** Irene Schaffner, Stefan Hofbauer, Michael Krutzler, Katharina F. Pirker, Marzia Bellei, Gerhard Stadlmayr, Georg Mlynek, Kristina Djinovic‐Carugo, Gianantonio Battistuzzi, Paul G. Furtmüller, Holger Daims, Christian Obinger

**Affiliations:** ^1^ Department of ChemistryDivision of BiochemistryBOKU – University of Natural Resources and Life SciencesMuthgasse 18A‐1190ViennaAustria; ^2^ Department for Structural and Computational BiologyMax F. Perutz LaboratoriesUniversity of ViennaViennaAustria; ^3^ Department of Microbiology and Ecosystem ScienceDivision of Microbial EcologyUniversity of ViennaViennaAustria; ^4^ Department of Life SciencesUniversity of Modena and Reggio Emilia41125ModenaItaly; ^5^ Department of Chemistry and GeologyUniversity of Modena and Reggio Emilia41125ModenaItaly; ^6^ Department of BiochemistryFaculty of Chemistry and Chemical TechnologyUniversity of Ljubljana1000LjubljanaSlovenia

## Abstract

It is demonstrated that cyanobacteria (both azotrophic and non‐azotrophic) contain heme *b* oxidoreductases that can convert chlorite to chloride and molecular oxygen (incorrectly denominated chlorite ‘dismutase’, Cld). Beside the water‐splitting manganese complex of photosystem II, this metalloenzyme is the second known enzyme that catalyses the formation of a covalent oxygen–oxygen bond. All cyanobacterial Clds have a truncated N‐terminus and are dimeric (i.e. clade 2) proteins. As model protein, Cld from *C* *yanothece* sp. PCC7425 (CCld) was recombinantly produced in *E* *scherichia coli* and shown to efficiently degrade chlorite with an activity optimum at pH 5.0 [*k*_cat_ 1144 ± 23.8 s^−1^, *K*_M_ 162 ± 10.0 μM, catalytic efficiency (7.1 ± 0.6) × 10^6^ M^−1^ s^−1^]. The resting ferric high‐spin axially symmetric heme enzyme has a standard reduction potential of the Fe(III)/Fe(II) couple of −126 ± 1.9 mV at pH 7.0. Cyanide mediates the formation of a low‐spin complex with *k*_on_ = (1.6 ± 0.1) × 10^5^ M^−1^ s^−1^ and *k*_off_ = 1.4 ± 2.9 s^−1^ (*K*_D_ ∼ 8.6 μM). Both, thermal and chemical unfolding follows a non‐two‐state unfolding pathway with the first transition being related to the release of the prosthetic group. The obtained data are discussed with respect to known structure–function relationships of Clds. We ask for the physiological substrate and putative function of these O_2_‐producing proteins in (nitrogen‐fixing) cyanobacteria.

**Abbreviations**
ABTS: 2,2′‐azinobis(3‐ethylbenzothiazoline‐6‐sulfonic acid)‐diammonium salt
CCld: chlorite dismutase from *Cyanothece* sp. PCC7425
Cld: chlorite dismutase
DSC: differential scanning calorimetry
DyP: dye‐decolourising peroxidase
*E*°': standard reduction potential
ECD: electronic circular dichroism
EPR: electron paramagnetic resonance
GdnHCl: guanidine hydrochloride
MALS: multi‐angle light scattering
NdCld: chlorite dismutase from ‘*Candidatus* Nitrospira defluvii’
NwCld: chlorite dismutase from *Nitrobacter winogradskyi*
PCRB: perchlorate reducing bacteria

## Introduction

Cyanobacteria are the only known bacteria capable of oxygenic (plant‐type) photosynthesis and are important model organisms for studies of the bioenergetics, evolution, and ecology of photosynthesis and aerobic respiration. They have accommodated both a photosynthetic electron transport chain and a respiratory transport chain within a single prokaryotic cell (Jones and Myers, 1963; Peschek *et al*., 2004; Paumann *et al*., 2005). Oxidation of water and release of O_2_ during oxygenic photosynthesis opened the era of an oxic atmosphere and also marked a turning point in the evolution of life on Earth, which not only provided novel ecological niches for organisms capable of aerobic respiration but also forced all life to either adapt to the presence of molecular oxygen or escape into the remaining anoxic habitats.

The water‐splitting manganese complex of photosystem II of cyanobacteria or plants was thought to be the only enzyme that forms a covalent O–O bond until the heme *b*‐containing oxidoreductase chlorite dismutase (Cld) was discovered in perchlorate‐reducing bacteria (PCRB). It catalyses the decomposition of chlorite (ClO_2_^−^) into chloride (Cl^−^) and molecular oxygen (O_2_) (van Ginkel *et al*., 1996). The proposed reaction mechanism of Cld with chlorite (Hofbauer *et al*., 2014a) comprises the formation of a [PorFe(III)]^+^ – ClO_2_^−^ adduct (1) which is rapidly followed by oxidation of the ferric heme to compound I [Por^●+^Fe(IV) = O]^+^ and HOCl/OCl^−^ as reaction intermediate (2). In a rebound mechanism, hypochlorite reacts with compound I which subsequently undergoes a two‐electron reduction to the ferric state, thereby releasing O = O and Cl^−^ (3).(1)${\left[\text{PorFe}\left(\text{III}\right)\right]}^{+}+{\text{ClO}}_{2}{}^{-}\to {\left[\text{PorFe}\left(\text{III}\right)\right]}^{+}\text{​}-\;{\text{ClO}}_{2}{}^{-}$
(2)${\left[\text{PorFe}\left(\text{III}\right)\right]}^{+}\text{​}-\;{\text{ClO}}_{2}{}^{-}\to {\left[{\text{Por}}^{•+}\text{Fe}\left(\text{IV}\right)=O\right]}^{+}+\text{HOCl}/{\text{OCl}}^{-}$
(3)${\left[{\text{Por}}^{•+}\text{Fe}\left(\text{IV}\right)\;=\;O\right]}^{+}+\text{HOCl}/{\text{OCl}}^{-}\to {\left[\text{PorFe}\left(\text{III}\right)\right]}^{+}+O_{2}+{\text{Cl}}^{-}$


PCRB are facultative anaerobes that can utilise perchlorate and chlorate as terminal electron acceptors in the absence of oxygen (Rikken *et al*., 1996). The product of this respiratory metabolism is chlorite, which exhibits strong cell‐damaging effects (Ueno H., 2000) and is detoxified by Cld to Cl^−^ and O_2_. The known functional (i.e. efficiently chlorite degrading) Clds can be divided into two phylogenetically distinct clades 1 and 2, which differ in subunit size and oligomerisation. So far, biochemical and structural research mainly focused on the pentameric or hexamerix clade 1 proteins (van Ginkel *et al*., 1996; Stenklo *et al*., 2001; Maixner *et al*., 2008; Streit and DuBois, 2008; Mehboob *et al*., 2009; Kostan *et al*., 2010), whereas only few studies addressed the dimeric clade 2 Clds from *Nitrobacter winogradskyi* and *Klebsiella pneumonia* MGH 78578 (Mlynek *et al*., 2011; Hofbauer *et al*., 2012a, 2012b; Celis *et al*., 2014).

Interestingly, proteins similar to Cld occur in a large number of deep‐branching bacterial and archaeal lineages. Their broad phylogenetic distribution indicates that these Cld‐like proteins represent an ancient protein family, which includes also the aforementioned two clades of functional Clds (Maixner *et al*., 2008; Kostan *et al*., 2010; Hofbauer *et al*., 2014c). The physiological roles of most functional Clds and Cld‐like proteins are elusive and many organisms containing them (e.g. *Cyanobacteria*) are not known to be PCRB or to produce chlorite as an intermediate of their metabolism.

The presence of (potentially O_2_‐evolving) Cld‐like proteins in cyanobacteria, and the importance of these organisms in the evolution of aerobic life, prompted us to analyse a cyanobacterial protein in detail. Here we report on the recombinant production of a Cld from the cyanobacterium *Cyanothece* sp. PCC7425 (CCld). The genus *Cyanothece* comprises a group of unicellular cyanobacteria which are morphologically as well as ecologically diverse, but all of them are capable of nitrogen fixation (Bandyopadhyay *et al*., 2011). Within this group, *Cyanothece* sp. PCC7425 has been shown to possess fundamentally different characteristics when compared with other *Cyanothece* strains. From a morphological point of view, the cells are smaller and more cylindrical and genetic analyses revealed modifications of its *nif* cluster. Nitrogen fixation could not be observed in the presence of oxygen, and only low levels of nitrogenase activity were detected under anaerobic conditions (Bandyopadhyay *et al*., 2011; 2013). There is evidence that *Cyanothece* sp. PCC7425 evolved (and is still evolving) independently from other *Cyanothece* strains, thereby losing its nitrogen‐fixing ability (Bandyopadhyay *et al*., 2011; 2013). In this paper we present the first comprehensive biochemical and biophysical characterisation of a cyanobacterial Cld in comparison with biochemical data of already characterised Clds from other microbial phyla. We demonstrate that cyanobacterial Clds exclusively belong to clade 2 of functional Clds and discuss putative physiological roles of these enzymes in cyanobacteria.

## Results

### Phylogeny of functional Clds and Cld‐like proteins

**Figure 1:**
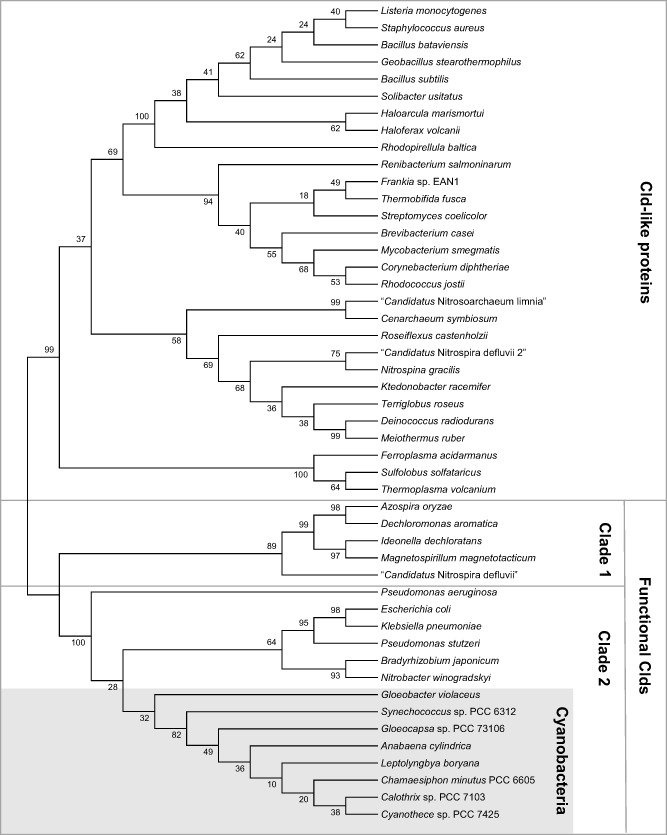
Reconstructed phylogenetic tree of 48 Cld‐like and functional Cld protein sequences. All sequences were extracted from NCBI database (www.ncbi.nlm.nih.gov). Multiple sequence alignments were performed using the MUSCLE algorithm (Edgar, 2004). The unrooted phylogenetic tree was reconstructed applying the maximum likelihood algorithm. The tree was tested by performing 1000 bootstrap replications. Sequence alignment as well as tree building were performed using MEGA6 (Tamura *et al*., 2013).

The multiple sequence alignment of 19 known functional Clds and 29 Cld‐like proteins was checked with regard to the alignment positions of two critical amino acid residues: a conserved proximal histidine residue, which serves as the fifth heme ligand in functional Clds and Cld‐like proteins (de Geus *et al*., 2009), and the catalytically important distal arginine that is conserved only in the known functional Clds (Kostan *et al*., 2010; Hofbauer *et al*., 2014b) (Fig. S1). In the obtained phylogenetic tree, the functional Clds and the Cld‐like proteins formed distinct clusters with high bootstrap support (Fig. 1). In accordance with former phylogenetic studies (Mlynek *et al*., 2011; Clark *et al*., 2013; Hofbauer *et al*., 2014c), the cluster of the functional Clds was further split into clades 1 and 2 (Fig. 1).

Interestingly, all cyanobacterial *cld* genes fell into clade 2 of the functional Clds and formed a separate sublineage within this clade (Fig. 1). A major difference between clades 1 and 2 is the drastically truncated N‐terminus of all clade 2 Clds (Hofbauer *et al*., 2014c). For example, the CCld monomer consists of only 182 amino acids whereas an NdCld subunit (clade 1) comprises 264 residues (Kostan *et al*., 2010). However, in the region around the active site, there is no obvious difference between the clades except an insertion of three amino acids in all clade 2 proteins, which is located N‐terminally of the conserved proximal histidine (Fig. S1). No *cld‐like* gene outside clade 2 of functional Clds was identified in any cyanobacterial genome.

Another difference between clade 1 and clade 2 Clds seems to be their localisation within the cell. Analysis of the N‐terminus suggests that all clade 2 Clds (as well as Cld‐like proteins) are located in the cytosol, whereas clade 1 enzymes are secreted to the periplasma (Table S2).

### Production, yield and Reinheitszahl (RZ) of recombinant CCld

Chlorite dismutase from *Cyanothece* sp. PCC7425 (CCld) was heterologously expressed in *Escherichia coli*. In detail, it could be produced as soluble intracellular protein. CCld was purified by affinity chromatography, as it was designed as Strep(II)‐tagged protein. After optimisation of the expression conditions in *E. coli*, a yield of approximately 3 mg protein per 10 g bacterial biomass was achieved. The purity of the protein was confirmed by sodium dodecyl sulphate–polyacrylamide gel electrophoresis and Western blotting with an anti‐Strep(II)‐tag antibody. Both the gel and the Western blot showed only one single band at an apparent molar mass of approximately 24 kDa (data not shown). This value for a monomer corresponds well to the theoretical molar mass of the dimeric native protein of 49.2 kDa (including one heme cofactor in each subunit).

**Figure 2:**
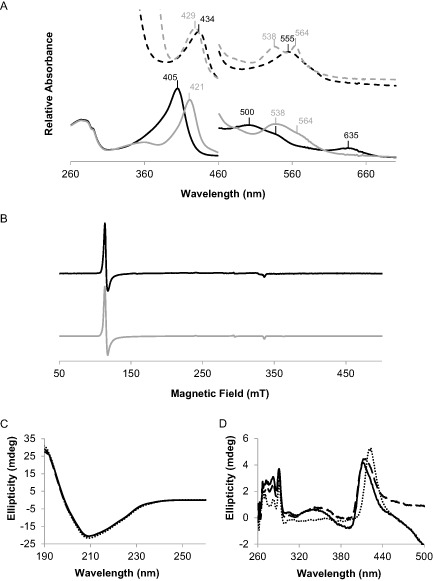
UV–vis, cw‐EPR and ECD spectra of chlorite dismutase from *C* *yanothece* sp. PCC7425 (CCld). A. UV–vis spectra of CCld recorded at pH 5.5. Spectra of the protein containing ferric high‐spin (absorption maximum at 405 nm) and low‐spin heme (absorption maximum at 421 nm) are shown as black and grey lines, respectively. Spectra of CCld containing ferrous high‐spin (absorption maximum at 434 nm) and low‐spin heme (absorption maximum at 429 nm) are shown as dotted lines in black and grey, respectively. As low‐spin ligand, cyanide was added. CCld was reduced by addition of 10 mM dithionite from a freshly prepared solution. B. cw‐EPR spectra of CCld at pH 5.5 and 10 K. The experimental and simulated spectra are shown as black and grey lines, respectively. C and D. ECD spectra of CCld in the far‐UV (C) and visible (D) region. Black bold lines show measurements at pH 5.5 (in 5 mM citrate phosphate buffer), black dashed lines show measurements at pH 7.0 (in 5 mM potassium phosphate buffer) and grey dotted lines show measurements at pH 10.0 (in 5 mM glycine buffer).

The UV–vis spectrum at pH 5.5 (Fig. 2A, black bold line) shows a Soret peak at 405 nm, a charge transfer (CT) band at 635 nm and Q bands at 500 nm (β) and 538 nm (α); all of them indicative of ferric high‐spin heme (Smulevich, 1996). The RZ, which is the ratio of the absorbance in the Soret region to the absorbance at 280 nm (A_405_/A_280_) and a measure of the heme content of the protein, was approximately 1.8 with slightly varying differences among batches. Altogether, these results show that recombinant CCld was produced as holoenzyme exhibiting a UV–vis spectrum that is very similar to that of other characterised functional Clds. Purified CCld aliquots with a concentration of 10 mg ml^−1^ were stored at −80°C until the protein was used for further analyses.

### Spectral properties of CCld

#### UV–vis spectra

In order to probe the activity of CCld we (i) converted the ferric high‐spin into the corresponding low‐spin form, (ii) reduced ferric high‐spin to ferrous high‐spin, and (iii) converted the ferrous high‐spin to the ferrous low‐spin form. Upon addition of the low‐spin ligand cyanide to native ferric high‐spin CCld, the Soret peak maximum shifted towards 421 nm. The CT band at 635 nm disappeared and the Q bands shifted from 500 nm (small shoulder at 538 nm) to a broad absorbance maximum around 538 nm (with shoulder at 564 nm) (Fig. 2A, grey bold line). Upon reduction of ferric to ferrous heme by addition of dithionite, the Soret maximum was red‐shifted to 434 nm and a Q band at 555 nm was observed (Fig. 2A, black dashed line). Treatment of the ferrous protein with cyanide caused a small shift of the Soret peak to 429 nm. A more pronounced change occurred in the visible region where two distinct Q bands were observed at 538 nm and 564 nm, respectively (Fig. 2A, grey dashed line).

#### EPR spectroscopy

To extend the picture of heme state and microenvironment, EPR experiments were performed. At 10 K and pH 5.5, the cw‐EPR spectrum of CCld (Fig. 2B) exhibits a typical axially symmetric coordinated ferric high‐spin signal characterised by xy‐plane symmetry, and a small contribution of a ferric low‐spin compound (Fig. 2B, black line). In order to determine high‐ and low‐spin contributions, the spectrum was simulated using the parameters listed in Table S3.

#### ECD spectroscopy

Electronic circular dichroism (ECD) spectra of CCld were recorded in the UV‐ as well as in the visible spectral region at pH values of 5.5, 7.0, and 10.0. A change of pH did not influence the shape of the almost congruent far‐UV spectra (Fig. 2C). CCld consists of approximately 30% alpha helices, 16.5% antiparallel and 8% parallel β‐sheets, 18.5% β‐turns and 27% random coil structure. These values did not change with a variation of pH. In the Soret region, a positive ellipticity signal was detected, which has previously been reported for other Clds (Hofbauer *et al*., 2012b) (Fig. 2D). A distinct pH‐dependent shift of the ellipticity of the Soret maximum was observed, indicating that pH does not influence the overall secondary structure of CCld, but influences the environment of the heme cofactor by modification of the ligand field and spin status.

### Oligomeric state of CCld analysed by SEC combined with MALS

**Figure 3:**
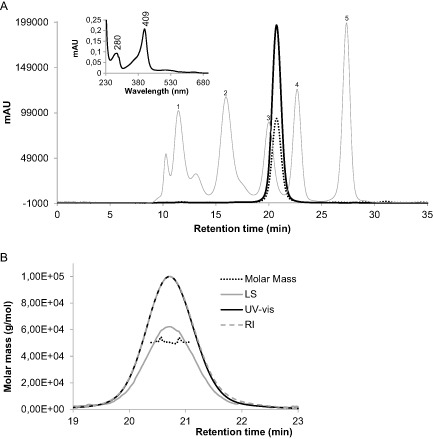
Investigation of molar mass and oligomeric state of chlorite dismutase from *C* *yanothece* sp. PCC7425 (CCld) by SEC and MALS. A. SEC elution profile of CCld followed by UV–vis detection at 412 nm (black bold line) and at 280 nm (black dotted line). The gel filtration standard (Biorad) is depicted as grey line (peak 1: thyroglobulin bovine, 670 000 Da; peak 2: γ‐globulin bovine, 158 000 Da; peak 3: ovalbumin chicken, 44 000 Da; peak 4: myoglobin horse, 17 000 Da; peak 5: vitamin B_12_, 1350 Da). The inset shows the corresponding UV–vis spectrum of the CCld fraction eluted at 20.7 min. B. Presentation of MALS analysis including light scattering (90° detection angle) (LS, grey line), UV–vis (black line) and refractive index (RI, grey dashed line) detection. The black dotted line shows the distribution of molar masses in the analysed CCld solution.

The only structurally characterised clade 2 Cld is NwCld from *Nitrobacter winogradskyi*. X‐ray crystallography experiments revealed a dimeric structure with two identical subunits (Mlynek *et al*., 2011). As CCld is also a clade 2 protein and thus expected to be of dimeric structure, we analysed its oligomeric state by size‐exclusion chromatography (SEC) combined with multi‐angle light scattering (MALS). CCld eluted from the column as one sharp single peak at a retention time of 20.7 min (Fig. 3A; the corresponding spectrum is depicted in the inset). MALS revealed an average size of 51 kDa which is highly similar to the theoretical mass of the dimeric protein of 49.2 kDa (Fig. 3B). CCld did not exhibit heterogeneity in its molar mass (Fig. 3B), and the average value could be calculated from all masses detected around the elution peak maximum (20.4–21.1 min).

### pH‐dependent chlorite degradation activity

**Figure 4:**
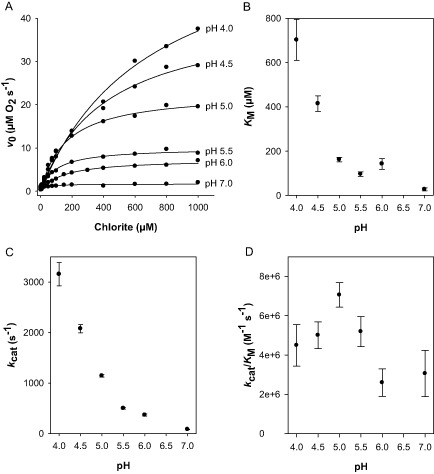
Polarographic measurement of chlorite degradation activity of chlorite dismutase from *C* *yanothece* sp. PCC7425 (CCld) and calculated kinetic parameters. A. Initial rate of oxygen generation (*v*_0_) plotted against chlorite concentrations. The reaction mixture contained 50 mM citrate phosphate buffer of various pH values (4.0–7.0) and chlorite in a concentration ranging from 2.5 to 1000 μM. The reaction was started by adding 20 nM CCld. Measurements were carried out under continuous stirring at 30°C. B–D. Kinetic parameters *K*_M_ (B), *k*_cat_ (C) and catalytic efficiency (D) at various pH values. At least two replicate measurements were performed for each assay.

Chlorite degradation activity of CCld and its pH dependence were measured polarographically with a Clark‐type electrode (initial generation of μM O_2_ per second at 30°C). The fitted reaction curves (*v*_0_ vs. ClO_2_^−^ concentration) are described by the equation *y* = *ax*/(*b* + *x*) (single rectangular hyperbola) (Fig. 4A). The total produced amount of O_2_ and the initial reaction velocity increased consistently with decreasing pH (Fig. 4A). Based on the obtained reaction curves, the catalytic parameters *k*_cat_, *K*_M_ and the catalytic efficiency *k*_cat_/*K*_M_ were calculated. Their change with pH is depicted in Fig. 4B–D, respectively. In contrast to other Cld activity studies (Hofbauer *et al*., 2014a), *k*_cat_ as well as *K*_M_ values increased drastically with a decreasing pH. Consequently, the catalytic efficiency did not vary pronouncedly with pH (Fig. 4D). However, pH 5.0 turned out to be the optimum for the chlorite‐degrading activity of CCld with a *k*_cat_ value of 1144 ± 23.8 s^−1^, a *K*_M_ of 162 ± 10.0 μM and a catalytic efficiency of (7.1 ± 0.6) × 10^6^ M^−1^ s^−1^. Reactions followed below pH 4.0 were independent from substrate concentrations and thus not meaningful, most probably due to immediate enzyme denaturation (data not shown).

### Probing alternative catalytic activities of CCld

Furthermore, we tested the ability of CCld to dismutate hydrogen peroxide by following the release of O_2_ by using a Clark‐type electrode. However, upon addition of 0.25 up to 40 mM H_2_O_2_ to 200 nM CCld at pH 7.0, no catalase activity but heme bleaching (irreversible loss of Soret absorbance) was observed (data not shown). Next we tested whether CCld is capable to catalyse the hydrogen peroxide‐mediated one‐electron oxidation of typical peroxidase substrates like 2,2′‐azinobis(3‐ethylbenzothiazoline‐6‐sulfonic acid)‐diammonium salt (ABTS) or guaiacol. Upon using 1 mM of ABTS or guaiacol and 100 nM CCld, a very low peroxidase activity was observed spectrophotometrically (pH 7.0 and 25°C) that depended on the H_2_O_2_ concentration (0.5–10 mM H_2_O_2_). Estimated *K*_M_ values (ABTS: 140 μM, guaiacol: 560 μM) and *k*_cat_ values (ABTS: 0.95 s^−1^; guaiacol: 0.23 s^−1^) were comparable with those reported for clade 1 Clds (Blanc *et al*., 2012).

Finally, we tested whether CCld is able to degrade alternative oxoanions of chlorine, bromine and iodine. However, upon addition of chlorate, bromate or iodate (0.1 mM–1 M) to 200 nM CCld, conversions of these (putative) substrates could not be observed (data not shown).

### Transient state kinetics of cyanide binding to CCld

**Figure 5:**
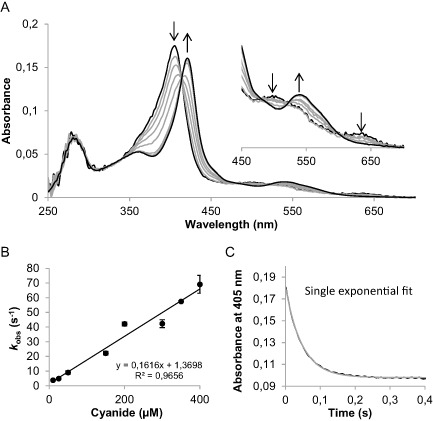
Transient‐state kinetics of binding of cyanide to chlorite dismutase of *C* *yanothece* sp. PCC7425 (CCld). A. Spectral changes during reaction of 2 μM CCld with 10 μM cyanide measured in conventional stopped‐flow mode. The first spectrum is the one of ferric CCld (absorption maximum at 406 nm), the second spectrum was recorded after 1.3 ms of mixing and the following spectra show the formation of the CCld‐cyanide low‐spin complex (absorption maximum at 421 nm). Arrows indicate directions of changes upon reaction. Measurements were performed in 50 mM phosphate buffer pH 7.0 at 25°C. B. Linear dependence of *k*_obs_ from cyanide concentrations. The apparent association rate constant *k*_on_ was obtained from the slope and the apparent dissociation rate constant *k*_off_ was calculated from the intercept. C. Typical time trace at 405 nm upon reaction of 2 μM CCld with 10 μM cyanide (single exponentially fitted).

Kinetic parameters of cyanide binding are frequently used to evaluate the accessibility of the active site of heme proteins. Binding of cyanide results in the conversion of high‐spin Fe(III) state (*S* = 5/2) to low‐spin Fe(III) state (*S* = 1/2). This reaction can be followed photometrically as it goes along with a red‐shift of the Soret maximum, in case of CCld from 405 to 421 nm. Distinct isosbestic points were observed at 414, 479, 520 and 596 nm (Fig. 5A). Monophasic binding of the ligand was monitored at 405 nm, and *k*_obs_ values were obtained by single‐exponential fitting of the curves (Fig. 5C). The apparent second‐order rate constant, *k*_on_ (1.6 ± 0.1) × 10^5^ M^−1^ s^−1^, was obtained from the slope of a linear plot of *k*_obs_ versus cyanide concentrations, whereas the apparent dissociation rate constant, *k*_off_ 1.4 ± 2.9 s^−1^, was calculated from the intercept of the plot. The ratio *k*_off_/*k*_on_ equals the dissociation constant, but as the standard error of the intercept is larger than *k*_off_, *K*_D_ of the cyanide complex can only be estimated to be around 8.6 μM.

### Redox chemistry of CCld

**Figure 6:**
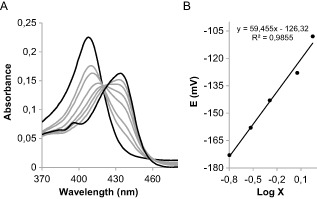
Spectroelectrochemical titration of the Fe(III)/Fe(II) redox couple of the high‐spin native form of chlorite dismutase from *C* *yanothece* sp. PCC7425 (CCld). A. Electronic absorption spectra of CCld at different applied potentials. Black lines represent fully oxidised (*A*_λox_^Max^ at 407 nm) and fully reduced form (*A*_λred_^Max^ at 434 nm). Titration was performed with 22 μM CCld in 100 mM potassium phosphate buffer pH 7.0 plus 100 mM NaCl. Additionally, 2 μM lumiflavine‐3‐acetate, methylene blue, phenazine methosulfate and indigo disulfonate were used as mediators. Temperature was held constant at 25°C. B. Corresponding Nernst plot, where X represents (*A*_λred_^Max^ − *A*_λred_)/(*A*_λox_^Max^ − *A*_λox_).

In order to determine the standard reduction potential (*E*°′) of CCld, spectroelectrochemical studies were performed. Figure 6A shows the fully oxidised and fully reduced (black lines, *A*_λox_^Max^ = 407 nm and *A*_λred_^Max^ = 434 nm) as well as the equilibrium spectra of CCld (grey lines) at six different applied potentials in the optical transparent thin‐layer spectroelectrochemical (OTTLE) cell (25°C, pH 7.0). Two clear isosbestic points at 420 and 460 nm were observed. From these data, we obtained a linear Nernst plot with a slope that is consistent with a one‐electron reduction process (Fig. 6B) (Millis *et al*., 1989; Dong *et al*., 1995; Battistuzzi *et al*., 2004). The standard reduction potential was calculated to be −126 ± 1.9 mV.

### pH‐dependent thermal stability monitored by DSC and ECD

**Figure 7:**
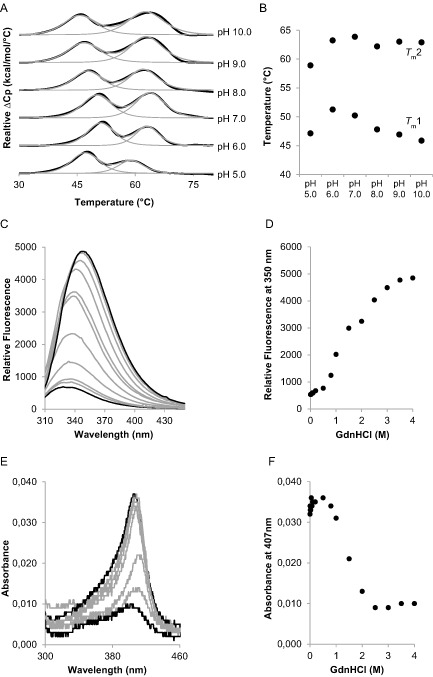
Investigation of thermal and conformational stability of chlorite dismutase from *C* *yanothece* sp. PCC7425 (CCld). A. DSC. Normalised thermograms of CCld over a pH range from 5.0 (bottom) to 10.0 (top). Black lines represent experimental data and grey lines show fit of endotherm to a non‐two‐state transition model. Measurements were performed in 50 mM citrate phosphate buffer (pH 5.0–7.0) and 50 mM Tris–HCl buffer (pH 8.0–10.0). Enzyme concentration was 20 μM. B. *T*_m_1 and *T*_m_2 values were plotted against pH values. C–F. Chemical denaturation mediated by addition of guanidine hydrochloride (GdnHCl) and measured by fluorescence and UV–vis spectroscopy. C. Increase of fluorescence with increasing concentration of GdnHCl. Excitation wavelength: 295 nm. Measurements were performed with 500 nM enzyme in 50 mM phosphate buffer pH 7.0. After addition of 0–4 M GdnHCl, samples were incubated at room temperature for 24 h. D. Relative fluorescence at 350 nm plotted against various GdnHCl concentrations. E. Decrease of absorbance with increasing concentration of GdnHCl. Measurements were performed with 500 nM enzyme in 50 mM phosphate buffer, pH 7.0. After addition of 0–4 M GdnHCl, samples were incubated at room temperature for 24 h. F. Absorbance at 407 nm plotted against various GdnHCl concentrations.

In order to investigate the unfolding behavior of CCld with regard to pH and temperature, we performed differential scanning calorimetry (DSC) as well as ECD experiments. Figure 7A shows the thermograms of CCld measured at pH 5.0–10.0. Two endotherms were observed upon melting of the enzyme, suggesting a non‐two‐state transition. Fitting of the curves to a non‐two‐state equilibrium unfolding model allowed the calculation of the *T*_m_1 (corresponding to the first transition) and *T*_m_2 (corresponding to the second transition) values (Fig. 7B). *T*_m_1 was highest at pH 6.0, i.e. 53.1°C, and continuously decreased with increasing pH to 44.9°C at pH 10.0. *T*_m_2 was highest at pH 7.0 (65°C) and did not deviate noticeably with increasing pH. In the acidic region, both *T*_m_1 and *T*_m_2 decreased drastically and no data could be obtained below pH 5.0 as the enzyme was denatured rapidly.

In order to assign the observed transitions to certain unfolding events, the pH dependence of thermal denaturation was also monitored by ECD (Fig. S2). We recorded melting curves of CCld at pH 5.5 (Fig. S2a,b), pH 7.0 (Fig. S2c,d) and pH 10.0 (Fig. S2e,f). At pH 7.0, two transitions were observed in both the far‐UV and the visible regions. The first transition with a calculated *T*_m_ value of 49°C was weakly pronounced in the far‐UV (Fig. S2c) but very distinct in the visible spectral region (Fig. S2d). This drastic change in ellipticity followed at 411 nm indicates that the first transition is due to release of the heme co‐factor. For the second transition (*T*_m_ = 63°C), the change of ellipticity intensities upon heating proceeds conversely. A significant loss of ellipticity at 210 nm (Fig. S2c) was observed, whereas only small changes in ellipticity were detected at 411 nm (Fig. S2d). From these observations, we conclude that the second transition reflects melting of the secondary structure elements of CCld.

### Chemical denaturation of CCld

In order to investigate the conformational stability of CCld, we chemically denatured the enzyme by stepwise addition of increasing concentrations of guanidine hydrochloride (GdnHCl). Subsequently, we (i) followed the increase of intrinsic tryptophan fluorescence (Fig. 7C) and (ii) the decrease of Soret absorbance in UV–vis (Fig. 7E) upon unfolding. The latter gives insight into active site events, whereas monitoring of fluorescence change yields information on denaturing processes of the overall protein. As with thermal unfolding, a non‐two‐state transition was observed (Fig. 7D and F). A closer look revealed that Soret absorbance at 407 nm was completely lost within 0.5 and 1.8 M GdnHCl in a two‐state transition (Fig. 7F). In contrast, the fluorescence intensity increased within 0.5 and 4 M GdnHCl with an intermediate step around 2 M GdnHCl (Fig. 7D). This clearly suggests that chemical unfolding follows the sequence native state → heme‐free intermediate → denatured state.

## Discussion

Among the 247 completely and partially sequenced cyanobacterial genomes (Table S1), eight (∼ 3–4%) contain a gene with high similarity to the dimeric CCld which we have characterised in this study, and to NwCld from *Nitrobacter winogradskyi* (Table 1). Both CCld and NwCld belong to clade 2 Clds that have a truncated N‐terminus (Mlynek *et al*., 2011). Sequence alignments (Fig. S1) suggest that the active site structure of CCld is highly similar to that of NwCld, which comprises a ferredoxin‐like fold with bound heme *b*. The distal ligand of the resting ferric state is an easily exchangeable water molecule, and the only important distal catalytic and charged residue is a mobile arginine. A histidine serves as proximal heme ligand. Due to hydrogen bonding with a glutamate, this amino acid has some imidazolate character (Mlynek *et al*., 2011). This structural similarity is also reflected by the UV–vis and EPR spectra of CCld, which resemble those of NwCld and suggest the presence of a dominating high‐spin ferric protein. Moreover, the standard reduction potential of −126 ± 1.9 mV at pH 7.0 is close to that of NwCld and also to that of clade 1 Clds (Hofbauer *et al*., 2012a; 2014c), underlining a highly similar heme ligation and environment.

**Table 1:**
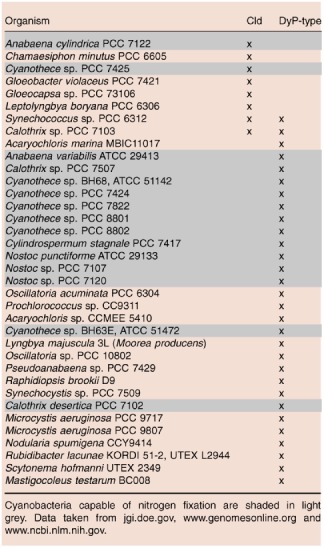
Cyanobacterial genomes possessing either *cld* or/and *dyp* genes

The catalytic efficiency of chlorite degradation by CCld slightly varies between (2–7) × 10^6^ M^−1^ s^−1^ within pH 4.0 and pH 7.0, whereas both *k*_cat_ and *K*_M_ decrease with increasing pH. Similar *k*_cat_/*K*_M_ values have been reported for Clds from other bacterial phyla (Hofbauer *et al*., 2014c). However, the pH dependence differs from that of pentameric (clade 1) Clds such as NdCld (from ‘*Candidatus* Nitrospira defluvii’), where a clear *k*_cat_/*K*_M_ peak was observed at pH 6.0 and the catalytic efficiency decreased with increasing pH (Hofbauer *et al*., 2014a). In CCld this pH dependence was less pronounced which might reflect some differences in the inhibition mechanism of clade 1 and clade 2 Clds. Recent studies demonstrated that transiently produced hypochlorite is responsible for the irreversible inhibition of pentameric NdCld, and that this inhibition becomes more pronounced with increasing pH values (Hofbauer *et al*., 2014a; Sündermann *et al*., 2014). It has been postulated that the catalytically important distal arginine has its p*K*_a_ around 6.5 (Streit *et al*., 2010), and due to highly similar active site architectures, this is most probably also true for other chlorite dismutases. The arginine has been shown to be very important to keep the reaction intermediate hypochlorite in the active site and prevent its escape and subsequent destruction of the enzyme. Due to deprotonation with increasing pH, the arginine becomes incapable of fulfilling this function. As a consequence, a decrease of chlorite degradation resulting from inhibition of the enzyme by escaped hypochlorite has been observed (Hofbauer *et al*., 2014a; Sündermann *et al*., 2014). Inhibition of NdCld can be suppressed by hypochlorite scavengers such as methionine or taurine (Hofbauer *et al*., 2014a), whereas the effect of these scavengers on CCld inhibition is small (unpublished results). Additionally, pentameric NdCld exhibits a higher thermal (melting point around 90°C) and conformational stability than dimeric Clds such as NwCld (*T*_m_ ∼ 50°C) (Hofbauer *et al*., 2012b) or CCld (Fig. 7).

In PCRB, Cld mediates the detoxification of chlorite that is an intermediate of (per)chlorate respiration. Recently, biological (per)chlorate reduction was detected in an ancient archaeal lineage, indicating that this metabolism could have been prevalent already at early stages of microbial evolution and might have released O_2_ into the atmosphere prior to the rise of oxygenic photosynthesis (Liebensteiner *et al*., 2013). However, cyanobacteria are not known to reduce (per)chlorate, and to our knowledge, no other source of endogenous chlorite in cyanobacteria has been described yet.

Recently, comprehensive proteomic profiling studies have been performed on *Cyanothece* sp. PCC7425 (Aryal *et al*., 2014). In the course of these experiments, chlorite dismutase could not be detected on protein level. However, conclusions from these findings should be drawn cautiously and in context with the applied cell cultivation conditions (i.e. supplemented with NaNO_3_, 30°C, continuous light, 7 days) (Aryal *et al*., 2014). Moreover, it has to be mentioned that the authors particularly emphasised on the difficult and complex proteome isolation and that it might be necessary to improve it in order to reach complete coverage of protein within this cyanobacterial species.

Presuming the expression of functional Clds in these organisms under certain (so far unknown) conditions, questions about the physiological roles of these enzymes are provoked. One possibility might be the detoxification of exogenous chlorite, but natural reservoirs of chlorite are rare on Earth and most chlorite present in the environment is of anthropogenic origin and appeared after the onset of industrialisation (Coates and Achenbach, 2004). Due to this short timeframe, anthropogenic chlorite pollution is unlikely to have been a selective force driving the evolution of two different Cld clades and their acquisition by phylogenetically distant microbial phyla (including *Cyanobacteria*). Moreover, a generally advantageous detoxifying function would suggest that clade 1 or 2 Clds were more widespread among environmental microorganisms than they seem to be according to currently available genome data. Whether bromite or iodite, which might be transient intermediates of bromate and iodate reduction, could be alternative substrates of Clds remains to be tested (in contrast to chlorite both bromite and iodite are very unstable and difficult to handle).

Iodate is naturally abundant in ocean waters and sediments (Bluhm *et al*., 2010), and the transformation of iodate to iodide by marine cyanobacteria has been described in the literature (Wong *et al*., 2002). However, neither iodate nor chlorate or bromate does act as substrate for CCld as demonstrated in this paper. The possibility that chlorite is formed intracellularly by reduction of chlorate remains. Nitrate reductases involved in anaerobic nitrate reduction were shown to reduce chlorate (Martínez‐Espinosa *et al*., 2015). It is neither known whether similar pathways occur in cyanobacteria nor if chlorate can be transported to the cytoplasma (where CCld is located).

Alternative activities of CCld such as catalase or peroxidase activities were shown to be negligible or very low. It is also noteworthy that an O_2_‐evolving activity of Cld (such as chlorite degradation) could be incompatible with N_2_ fixation by the highly oxygen‐sensitive enzyme nitrogenase, which is found in many cyanobacteria including some that also possess Cld (Table 1).

Cld‐like proteins from Gram‐positive bacteria *Bacillus subtilis*, *Mycobacterium tuberculosis*, *Listeria monocytogenes* and *Staphylococcus aureus* have been reported to play a role in heme biosynthesis (Dailey *et al*., 2010; 2015; Mayfield *et al*., 2013; Hofbauer *et al*., 2015). A function of the cyanobacterial Clds in this or another housekeeping pathway seems unlikely, because only a minority (4%, see also discussion earlier) of all sequenced cyanobacterial genomes contain a *cld* gene.

The efficient chlorite‐degrading clade 1 and 2 Clds, the Cld‐like proteins and the dye‐decolourising peroxidases (DyPs) form a structural superfamily, suggesting common phylogenetic roots of these enzymes (Hofbauer *et al*., 2014c). The DyPs reduce hydrogen peroxide using a broad variety of electron donors. They possess a conserved distal aspartate residue in addition to the Arg found in clade 1 and 2 Clds. Genes encoding DyPs occur in ∼ 12–13% of the sequenced cyanobacterial genomes, but the biological roles of the respective enzymes are unknown. Table 1 demonstrates that (with the exception of two representatives) cyanobacteria contain either Clds or DyP‐type peroxidases, which might indicate a similar function(s). In any case, the occurrence of DyPs broadens the repertoire of heme‐containing and hydrogen peroxide reducing cyanobacterial enzymes now including bifunctional catalase–peroxidase, monofunctional catalase, novel bacterial peroxidases with homology to mammalian counterparts and DyPs (Auer et al., 2013; Bernroitner *et al*., 2009).

Summing up, this paper presents a comprehensive biochemical and biophysical characterisation of a dimeric chlorite dismutase. It is the truncated cytosolic Cld from clade 2 and the first characterised cyanobacterial representative. The recombinant heme enzyme from *Cyanothece* sp. PCC7425 efficiently decomposes chlorite to chloride and molecular oxygen and is less sensitive to inactivation by the transiently produced intermediate hypochlorous acid compared with pentameric clade 1 Clds. UV–vis‐, ECD‐ and EPR‐ spectroscopy demonstrated the occurrence of high‐spin well‐folded heme *b* active site architecture with typical accessibility and affinity of cyanide. Only 4% of cyanobacterial genomes contain *cld* genes, whereas in about 12–13% of genome genes encoding DyPs are found. Cld‐like proteins do not occur in cyanobacteria.

## Experimental procedures

### Phylogenetic analysis and tree building

Analysis of sequenced cyanobacterial genomes was performed using public databases (http://www.ncbi.nlm.nih.gov, http://www.genomesonline.org). For the reconstruction of a phylogenetic tree, the amino acid sequences of 29 Cld‐like proteins and of 19 functional Clds were extracted from the NCBI database and aligned by using the MUSCLE algorithm (Edgar, 2004) with the following settings: gap open −2.9, gap extend 0, hydrophobicity multiplier 1.2, maximum iterations 8, clustering method UPGMB. A phylogenetic tree was then calculated by applying the maximum likelihood algorithm with the following parameters: Jones–Taylor–Thornton model, gamma distribution set to 3, complete deletion of gaps/missing data, and 1000 bootstrap iterations. Sequence alignment and tree reconstruction were performed using the MEGA6 software (Tamura *et al*., 2013).

Analysis of putative signal sequences was performed with SignalP 4.1 (Petersen *et al*., 2011), TatP 1.0 (Bendtsen *et al*., 2005b) and Secretome 2.0 (Bendtsen *et al*., 2005a) (http://www.expasy.org).

### Cloning, heterologous expression and purification of CCld

A DNA fragment containing the full‐length coding region (182 amino acids) of Cld from *Cyanothece* sp. PCC7425 (accession no. YP_002482168.1) (CCld) was amplified by polymerase chain reaction using the newly designed primers CCldF (5′‐ACAACGGGGGGATCCAAACAATCGTTATTCTTTCATCGGCGGTCGCAC‐3′) and CCldR (5′‐CTAAACAAGCGGCCGCTTACAGGCGTTTCAGCCAAACTTC‐3′) (Sigma Aldrich). The plasmid vector pJExpress401 containing the desired chlorite dismutase sequence was used as a template. The amplicon was cloned into the pET‐52b + expression vector (Merck Millipore, Hessen, Germany) for the subsequent production of an N‐terminally Strep(II)‐tagged fusion protein. Successful cloning was confirmed by sequencing from T7prom and T7term (LGC Genomics, Germany).

Recombinant CCld was expressed in *E. coli* BL21 Gold (DE3) cells (Agilent Technologies, Santa Clara, CA, USA). An expression condition screening has been performed in order to find optimal protein production parameters. In particular, we probed different inducer concentrations (0.1, 0.5 and 1 mM IPTG), time points of induction (OD_600_: 0.6–1.0), expression temperatures (16–30°C) and optimal duration of expression (cell harvest after 6–20 h). The respective samples were tested for target protein content in the soluble fraction by Western blotting using an anti‐Strep(II)‐tag antibody.

Cells were cultivated in Luria–Bertani medium supplemented with ampicillin (100 μg ml^−1^). An overnight culture was used for inoculation at a dilution ratio of 1:100. The culture was grown at 37°C under agitation (180 rpm). Based on the screening results, cells were grown to an optical density at 600 nm (OD_600_) of 0.6, then hemin chloride (50 μg ml^−1^) and isopropyl‐β‐D‐thiogalactopyranoside (IPTG) (120 μg ml^−1^) were added to the supernatant. For protein expression, the temperature was reduced to 16°C. After 20 h, the culture was centrifuged (5000 *g*, 20 min, 4°C) and the obtained cell pellet was either processed immediately (see discussion later) or stored at −80°C.

The cell pellet was resuspended in 50 mM HEPES pH 7.4, 5% glycerol, 0.5% Triton X‐100, 0.5 mM EDTA and 1 mM phenylmethylsulfonylfluoride. Cells were lysed by sonication and cell debris was removed by centrifugation (17 000 *g*, 30 min, 4°C). Subsequently, the supernatant containing soluble CCld was filtrated (0.45 μm) and loaded onto a StrepTrap HP 5 ml column (GE Healthcare, Vienna, Austria) equilibrated with 20 mM HEPES pH 7.4 and 2% glycerol. Elution was achieved by using 20 mM HEPES pH 7.4, 2% glycerol and 1 mM d‐desthiobiotin. The eluted protein was screened photometrically and fractions containing CCld were pooled, concentrated and desalted using a 10 kDa Amicon Ultra Centrifugal Filter (Merck Millipore, Hessen, Germany). Aliquots (∼ 10 mg ml^−1^) were stored in 50 mM potassium phosphate buffer pH 7.0, at −80°C.

### UV–vis spectroscopy

UV–vis spectra were recorded on an Agilent 8453 diode array spectrophotometer (Agilent Technologies, Santa Clara, CA, USA) and on a U‐3900 spectrophotometer (Hitachi, Mannheim, Germany). Reduced spectra were generated by addition of O_2_‐free sodium dithionite from a freshly prepared stock solution. The molar extinction coefficient of heme (ϵ_409nm_ = 100 000 M^−1^ cm^−1^) was used to determine the enzyme concentration.

### EPR spectroscopy

Electron paramagnetic resonance spectroscopy was performed on a Bruker EMX continuous wave (cw) spectrometer, operating at X‐band (9 GHz) frequencies. The instrument was equipped with a high sensitivity resonator and an Oxford Instruments ESR900 helium cryostat (Oxford Instruments, Oxfordshire, United Kingdom) for low‐temperature measurements. Spectra were recorded under non‐saturating conditions using 2 mW microwave power, 100 kHz modulation frequency, 1 mT modulation amplitude and 20 ms conversion time, 20 ms time constant and 4096 points. For the measurements, 100 μl samples of 50 μM recombinant CCld were prepared in 50 mM MES (2‐(N‐morpholino)ethanesulfonic acid) buffer pH 5.5, transferred into Wilmad quartz tubes (3 mm inner diameter) and flash frozen in liquid nitrogen. In order to remove O_2_, the tubes were flushed with argon while the sample was kept frozen on dry ice. Measurements were performed at 10 K. High‐spin and low‐spin spectra were simulated using the software EasySpin (Stoll and Schweiger, 2006) and consist of a weighted sum of simulations of the individual high‐spin and low‐spin compounds. The rhombicity was obtained from *g_x_* ^eff^ and *g_y_* ^eff^ (Peisach *et al*., 1971) and the relative intensities were calculated on the basis of the simulations.

### ECD spectroscopy

Electronic circular dichroism spectroscopy was performed using Chirascan (Applied Photophysics, Leatherhead, UK). First, the instrument was flushed with nitrogen at a flow rate of 5 l min^−1^. Then, ECD spectra were recorded at room temperature in the far‐UV region (i.e. 180–260 nm) and in the visible region (i.e. 260–500 nm) using 10 μM CCld. Used buffers were 5 mM citrate phosphate, 5 mM potassium phosphate and 5 mM glycine for pH 5.5, 7.0 and 10.0, respectively. The path length was 10 mm for the visible region and 1 mm for far‐UV, spectral band width was 3 nm and scan time per point was 10 s.

The same settings were used for monitoring temperature‐mediated unfolding between 20 and 80°C with a heat rate of 1°C min^−1^. Far‐UV and visible ECDs were performed at 210 nm and at the Soret maximum, respectively. The obtained data were processed using Pro‐Data Viewer provided by Applied Photophysics. The fraction of unfolded protein (α) at certain temperatures was calculated according to the formula α = (ϴ_N_ − ϴ)/(ϴ_N_ − ϴ_U_), where ϴ_N_ is the ellipticity in mdeg at 210/411 nm of the native protein, ϴ_U_ represents the ellipticity at 210/411 nm of the unfolded protein and ϴ is the ellipticity at 210/411 nm of the protein at distinct temperatures.

### SEC and MALS

Size‐exclusion chromatography combined with MALS was performed to determine the molar mass and oligomeric state of CCld. HPLC (Shimadzu Prominence LC20) (Shimadzu Europe GmbH, Duisburg, Germany) was equipped with MALS (WYATT Heleos Dawn8+plus QELS; software ASTRA 6), refractive index detector (RID‐10A, Shimadzu) and a diode array detector (SPD‐M20A, Shimadzu). The column (Superdex 200 10/300 GL, GE Healthcare) had a particle size of 13 μm. Experiments were performed at a flow rate of 0.75 ml min^−1^ and the injected protein amount was 50 μg. As running buffer, Dulbecco PBS plus 200 mM NaCl was used.

### Steady‐state kinetics

Chlorite degradation activity of CCld was measured polarographically by using a Clark‐type oxygen electrode (Oxygraph plus, Hansatech Instruments Ltd, Norfolk, United Kingdom). Reactions were followed at 30°C and the temperature was held constant using a water bath. The electrode was equilibrated to 100% O_2_ saturation by flushing with air and to 0% O_2_ saturation by flushing with N_2_ until plateaus were reached in order to derive an offset and calibration factor. Reactions were carried out in O_2_‐free 50 mM citrate phosphate buffer with pH values ranging from 4.0 to 7.0. NaClO_2_ was applied in concentrations ranging from 2.5 to 1000 μM. Reactions were started by addition of 20 nM CCld. Molecular oxygen production rates (μM O_2_ s^−1^) were calculated from the initial linear time traces and plotted against chlorite concentrations.

Putative chlorate, bromate and/or iodate degradation activity was tested using the same method. The instrument was prepared as described earlier and the measurements were conducted in 50 mM citrate phosphate buffer with pH 5.0 and 7.0. Chlorate, bromate and iodate were applied in a concentration range between 0.1 mM and 1 M depending on the solubility of the respective sodium or potassium salt. About 200 nM CCld were used.

Furthermore, Clark‐type electrode measurements were performed to test hydrogen peroxide dismutation activity of CCld. The experiments were performed at pH 5.0 and 7.0 using 50 mM citrate phosphate buffer. H_2_O_2_ was used in a concentration range between 0.25 and 40 mM and 200 nM CCld was used.

Peroxidase activity was tested photometrically using the one‐electron donors guaicol and ABTS. Measurements were performed in 50 mM citrate phosphate buffer pH 5 and 7. 1 mM ABTS/guaiacol were applied, and H_2_O_2_ concentration was varied from 0.5 to 10 mM. For each experiment, 100 nM CCld were used to start the reaction. Rates of guaiacol or ABTS oxidation were calculated by following the absorbance increase at 470 nm (ϵ_470_ = 26.6 mm^−1^ cm^−1^) (Doerge *et al*., 1997) and 414 nm (ϵ_414_ = 36 mm^−1^ cm^−1^) (Childs and Bardsley, 1975), respectively.

### Stopped‐flow UV–vis spectroscopy

Cyanide binding studies were carried out on a stopped‐flow apparatus (model SX‐18MV, Applied Photophysics) equipped for both conventional and sequential measurements. The optical quartz cell had a pathlength of 10 mm and a volume of 20 μl. All experiments were conducted at 25°C. The fastest time for mixing two solutions and recording the first data point was 1 ms. Binding of cyanide to CCld was investigated using a diode array detector (Applied Photophysics). This allowed the acquisition of a set of time‐resolved spectra from a single stopped‐flow drive. In order to record time traces, conventional stopped‐flow mode was applied and decrease in absorbance at 405 nm was monitored using a photomultiplier (Applied Photophysics). For a typical measurement, 2 μM CCld and sodium cyanide concentrations from 10 to 400 μM were applied (50 mM potassium phosphate buffer, pH 7.0). At least two measurements were performed for each ligand concentration. From the plot of the pseudo‐first‐order rate constant *k*_obs_ versus cyanide concentration, the apparent second‐order rate constant, *k*_on_, was obtained.

### Spectroelectrochemistry

The standard reduction potential, *E*°′, of the Fe(III)/Fe(II) couple was determined using a homemade OTTLE cell (Battistuzzi *et al*., 2001; 2006; 2010). The three‐electrode configuration consisted of a gold minigrid working electrode (Buckbee–Mears), a homemade Ag/AgCl/KCl_sat_ microreference electrode, separated from the working solution by a Vycor set, and a platinum wire as the counter electrode (Battistuzzi *et al*., 2001; 2006; 2010). The reference electrode was calibrated against a saturated calomel (HgCl) electrode before each set of measurements. All potentials are referenced to the standard hydrogen electrode (242 mV). Potentials were applied across the OTTLE cell with an Amel model 2053 potentiostat/galvanostat. A constant temperature was maintained by a circulating water bath and the OTTLE cell temperature was monitored with a copper‐costan microthermo‐couple. UV–vis spectra were recorded using a Varian Cary C50 spectrophotometer (Agilent Technologies, Santa Clara, CA, USA). The OTTLE cell was flushed with argon gas to establish an oxygen‐free environment in the cell (Battistuzzi *et al*., 2001; 2006; 2010). Conditions: 22 μM CCld in 100 mM potassium phosphate buffer, pH 7.0, plus 100 mM NaCl. Additionally, 2 μM lumiflavine‐3‐acetate, methylene blue, phenazine methosulfate and indigo disulfonate were used as mediators.

### DSC

Differential scanning calorimetry experiments were carried out on a VP‐capillary DSC microcalorimeter from Microcal (GE Healthcare, Vienna, Austria) (cell volume: 137 μl) controlled by the VP‐viewer program and equipped with an autosampler for 96 well plates. The heating scan rate was programmed to 60°C h^−1^ and CCld samples were analysed over a temperature range of 20–85°C. Cell pressure was approximately 60 psi (4.136 bar). CCld was applied as 20 μM solution in 50 mM citrate phosphate buffer with pH values ranging from 4.0 to 7.0 and in 50 mM Tris–HCl buffer, pH 8.0–10.0. For data analysis and processing, the Microcal Origin 7 software was used. First, buffer baselines were subtracted from the obtained data. Subsequently, normalisation for protein concentration was performed. Data were fitted to a non‐two‐state equilibrium unfolding model by the Lavenberg/Marquardt non‐linear least squares method.

### Chemical unfolding

In order to probe chemical denaturation of CCld, samples were incubated with increasing concentrations of guanidine hydrochloride (GdnHCl) and unfolding was monitored by following changes in the emission of intrinsic tryptophan fluorescence as well as changes in the absorbance in the Soret band region.

For these experiments, 500 nM CCld in 50 mM potassium phosphate buffer, pH 7.0, was incubated with increasing concentrations of GdnHCl (0–5 M) over night and at room temperature. Total assay volume was 2 ml. UV–vis spectra were recorded on a Hitachi U‐3900 spectrophotometer and fluorescence spectra were measured on a Hitachi F‐7000 Fluorescence spectrophotometer. Both were equipped with a thermostatic cell holder for quartz cuvettes of 10 mm path length. Instrumental parameters for fluorescence measurements were set as follows: PMT voltage was 700 V, excitation wavelength was at 295 nm, excitation and emission bandwidth at 5 nm and scan speed was 60 nm min^−1^. The fraction of unfolded protein (α) at certain GdnHCl concentrations was calculated according to the formula α = (*F*_N_ − *F*)/(*F*_N_ − *F*_U_), where *F*_N_ is the fluorescence intensity at 350 nm of the native protein, *F*_U_ represents the fluorescence intensity at 350 nm of the unfolded protein and *F* is the fluorescence intensity at 350 nm of the protein after incubation at certain GdnHCl concentrations.

UV–vis spectra were recorded with a scan rate of 300 nm s^−1^. The fraction of unfolded protein (α) at certain GdnHCl concentrations was calculated according to the formula α = (*A*_N_ − *A*)/(*A*_N_ − *A*_U_), where *A*_N_ is the absorbance at the Soret maximum of the native protein, *A*_U_ represents the absorbance at the Soret maximum of the unfolded protein and *A* is the absorbance at the Soret maximum of the protein after incubation at certain GdnHCl concentrations.

## Supporting information


Supporting information
