# Supplementary material for: Dimeric chlorite dismutase from the nitrogen‐fixing cyanobacterium C yanothece sp. PCC7425
Source: Mol Microbiol. 2015 Apr 6;96(5):1053–68. doi: 10.1111/mmi.12989 (PMC4973843; doi:10.1111/mmi.12989)
Supplement: Supplementary file 1 — Supporting information [file MMI-96-1053-s001.docx]

**SUPPLEMENTARY MATERIAL**

**Dimeric chlorite dismutase from the nitrogen-fixing cyanobacterium *Cyanothece* sp*.* PCC7425**

**Irene Schaffner^1^, Stefan Hofbauer^1,2^, Michael Krutzler^1^, Katharina F. Pirker^1^, Marzia Bellei^5^, Gerhard Stadlmayr^1^, Georg Mlynek^2^, Kristina Djinovic-Carugo^2,3^, Gianantonio Battistuzzi^6^, Paul G. Furtmüller^1^, Holger Daims^4^ and Christian Obinger^1^***

^1^Department of Chemistry, Division of Biochemistry, BOKU – University of Natural Resources and Life Sciences, Muthgasse 18, A-1190 Vienna

^2^Department for Structural and Computational Biology, Max F. Perutz Laboratories, University of Vienna, A-1030 Vienna, Austria

^3^Department of Biochemistry, Faculty of Chemistry and Chemical Technology, University of Ljubljana, 1000 Ljubljana, Slovenia

^4^Department of Microbiology and Ecosystem Science, Division of Microbial Ecology, University of Vienna, A-1090 Vienna, Austria

^5^Department of Life Sciences, University of Modena and Reggio Emilia, 41125 Modena, Italy

^6^Department of Chemistry and Geology, University of Modena and Reggio Emilia, 41125 Modena, Italy

*corresponding author: Christian Obinger, email: [christian.obinger@boku.ac.at](mailto:christian.obinger@boku.ac.at), phone: +43-1-47654-6073, fax: +43-1-47654-6050

**Table S1.** List of completely or partially sequenced cyanobacterial genomes (as of 28/07/2014). Data taken from [jgi.doe.gov](http://jgi.doe.gov/) and [www.genomesonline.org](http://www.genomesonline.org/).

| **Organism** | **Sequencing Status GOLD 28/07/2014** |
| --- | --- |
| *Acaryochloris marina* MBIC11017 | Complete |
| *Acaryochloris* sp. CCMEE 5410 | Complete |
| *Anabaena cylindrica* PCC 7122 | Complete |
| *Anabaena* sp. 90 | Complete |
| *Anabaena* sp. PCC 7108 | Complete |
| *Anabaena variabilis* ATCC 29413 | Complete |
| *Arthrospira maxima* CS-328 | Complete |
| *Arthrospira platensis* C1 | Complete |
| *Arthrospira platensis* NIES-39 | Complete |
| *Arthrospira platensis* Paraca | Complete |
| *Arthrospira* sp. PCC 8005 | Complete |
| *Calothrix desertica* PCC 7102 | Complete |
| *Calothrix* sp. PCC 6303 | Complete |
| *Calothrix* sp. PCC 7103 | Complete |
| *Calothrix* sp. PCC 7507 | Complete |
| *Chamaesiphon minutus* PCC 6605 | Complete |
| *Chlorogloeopsis fritschii* PCC 6912 | Complete |
| *Chlorogloeopsis* sp. PCC 9212 | Complete |
| *Chroococcidiopsis* sp. PCC 6712 | Complete |
| *Chroococcidiopsis thermalis* PCC 7203 | Complete |
| *Crinalium epipsammum* PCC 9333 | Complete |
| *Crocosphaera watsonii* WH 8501 | Complete |
| *Crocosphaera watsonii* WH0003 | Complete |
| *Cyanobacteria* JGI 0000014-E08 | Complete |
| *Cyanobacterium aponinum* PCC 10605 | Complete |
| *Cyanobacterium* PCC 7702 | Complete |
| *Cyanobacterium* sp. ESFC-1 | Complete |
| *Cyanobacterium* sp. UCYN-A | Complete |
| *Cyanobacterium stanieri* PCC 7202 | Complete |
| *Cyanobium gracile* PCC 6307 | Complete |
| *Cyanobium* sp. PCC 7001 | Complete |
| *Cyanothece* sp. BH63E, ATCC 51472 | Complete |
| *Cyanothece* sp. BH68, ATCC 51142 | Complete |
| *Cyanothece* sp. CCY 0110 | Complete |
| *Cyanothece* sp. PCC 7424 | Complete |
| *Cyanothece* sp. PCC 7425 | Complete |
| *Cyanothece* sp. PCC 7822 | Complete |
| *Cyanothece* sp. PCC 8801 | Complete |
| *Cyanothece* sp. PCC 8802 | Complete |
| *Cylindrospermopsis raciborskii* CS-505 | Complete |
| *Cylindrospermum stagnale* PCC 7417 | Complete |
| *Dactylococcopsis salina* PCC 8305 | Complete |
| *Dolichospermum circinale* ACBU02 | Complete |
| *Fischerella muscicola* PCC 7414 | Complete |
| *Fischerella muscicola* SAG 1427-1 | Complete |
| *Fischerella* sp. JSC-11 | Complete |
| *Fischerella* sp. PCC 9339 | Complete |
| *Fischerella* sp. PCC 9431 | Complete |
| *Fischerella* sp. PCC 9605 | Complete |
| *Fischerella thermalis* PCC 7521 | Complete |
| *Fremyella diplosiphon* UTEX 481 | Complete |
| *Geitlerinema* sp. PCC 7105 | Complete |
| *Geitlerinema* sp. PCC 7407 | Complete |
| *Geminocystis herdmanii* PCC 6308 | Complete |
| *Gloeobacter violaceus* PCC 7421 | Complete |
| *Gloeocapsa* sp. PCC 73106 | Complete |
| *Gloeocapsa* sp. PCC 7428 | Complete |
| *Halothece* sp. PCC 7418 | Complete |
| *Leptolyngbya boryana* PCC 6306 | Complete |
| *Leptolyngbya* sp. PCC 6406 | Complete |
| *Leptolyngbya* sp. PCC 7375 | Complete |
| *Leptolyngbya sp.* PCC 7376 | Complete |
| *Leptolyngbya valderiana* BDU 20041 | Complete |
| *Lyngbya majuscula* 3L (*Moorea producens*) | Complete |
| *Lyngbya* sp. CCY 8106 | Complete |
| *Mastigocladopsis repens* PCC 10914 | Complete |
| *Mastigocoleus testarum* BC008 | Complete |
| *Microchaete* sp. PCC 7126 | Complete |
| *Microcoleus chthonoplastes* PCC 7420 | Complete |
| *Microcoleus* sp. PCC 7113 | Complete |
| *Microcoleus vaginatus* FGP-2 | Complete |
| *Microcystis aeruginosa* NIES-843 | Complete |
| *Microcystis aeruginosa* PCC 7806 | Complete |
| *Microcystis aeruginosa* PCC 7941 | Complete |
| *Microcystis aeruginosa* PCC 9432 | Complete |
| *Microcystis aeruginosa* PCC 9443 | Complete |
| *Microcystis aeruginosa* PCC 9701 | Complete |
| *Microcystis* *aeruginosa* PCC 9717 | Complete |
| *Microcystis aeruginosa* PCC 9806 | Complete |
| *Microcystis aeruginosa* PCC 9807 | Complete |
| *Microcystis aeruginosa* PCC 9808 | Complete |
| *Microcystis aeruginosa* PCC 9809 | Complete |
| *Microcystis aeruginosa* SPC777 | Complete |
| *Microcystis aeruginosa* TAIHU98 | Complete |
| *Microcystis* sp. T1-4 | Complete |
| *Nodosilinea nodulosa* PCC 7104 | Complete |
| *Nodularia spumigena* CCY9414 | Complete |
| *Nostoc azollae* 708 | Complete |
| *Nostoc punctiforme* ATCC 29133 | Complete |
| *Nostoc* sp. PCC 7107 | Complete |
| *Nostoc* sp. PCC 7120 | Complete |
| *Nostoc* sp. PCC 7524 | Complete |
| *Oscillatoria acuminata* PCC 6304 | Complete |
| *Oscillatoria formosa* PCC 6407 | Complete |
| *Oscillatoria nigro-viridis* PCC 7112 | Complete |
| *Oscillatoria* sp. PCC 10802 | Complete |
| *Oscillatoria* sp. PCC 6506 | Complete |
| *Oscillatoriales* sp. JSC-1 | Complete |
| *Oscillatoriales* sp. JSC-12 | Complete |
| *Planktothrix agardhii* | Complete |
| *Planktothrix rubescens* NIVA-CYA 98 | Complete |
| *Pleurocapsa* sp. PCC 7319 | Complete |
| *Pleurocapsa* sp. PCC 7327 | Complete |
| *Prochlorococcus marinus* | Complete |
| *Prochlorococcus marinus* AS9601 | Complete |
| *Prochlorococcus marinus* CCMP 1375 | Complete |
| *Prochlorococcus marinus* CCMP 1986 | Complete |
| *Prochlorococcus marinus* MIT 9211 | Complete |
| *Prochlorococcus marinus* MIT 9215 | Complete |
| *Prochlorococcus marinus* MIT 9301 | Complete |
| *Prochlorococcus marinus* MIT 9303 | Complete |
| *Prochlorococcus marinus* MIT 9312 | Complete |
| *Prochlorococcus marinus* MIT 9313 | Complete |
| *Prochlorococcus marinus* MIT 9515 | Complete |
| *Prochlorococcus marinus* MIT9202 | Complete |
| *Prochlorococcus marinus* NATL1A | Complete |
| *Prochlorococcus marinus* NATL2A | Complete |
| *Prochlorococcus* sp. CC9311 | Complete |
| *Prochlorococcus* sp. CC9605 | Complete |
| *Prochlorococcus* sp. CC9902 | Complete |
| *Prochlorococcus* sp. W10 | Complete |
| *Prochlorococcus* sp. W11 | Complete |
| *Prochlorococcus* sp. W12 | Complete |
| *Prochlorococcus* sp. W2 | Complete |
| *Prochlorococcus* sp. W3 | Complete |
| *Prochlorococcus* sp. W4 | Complete |
| *Prochlorococcus* sp. W5 | Complete |
| *Prochlorococcus* sp. W6 | Complete |
| *Prochlorococcus* sp. W7 | Complete |
| *Prochlorococcus* sp. W8 | Complete |
| *Prochlorococcus* sp. W9 | Complete |
| *Prochlorococcus* sp. WH 7803 | Complete |
| *Prochlorococcus* sp. WH8102 | Complete |
| *Prochloron didemni* P1-Palau | Complete |
| *Prochlorothrix hollandica* PCC 9006 | Complete |
| *Pseudanabaena* sp. PCC 6802 | Complete |
| *Pseudanabaena* sp. PCC 7367 | Complete |
| *Pseudoanabaena* sp. PCC 7429 | Complete |
| *Raphidiopsis brookii* D9 | Complete |
| *Richelia intracellularis* HH01 | Complete |
| *Rivularia* sp. PCC 7116 | Complete |
| *Rubidibacter lacunae* KORDI 51-2, UTEX L2944 | Complete |
| *Scytonema hofmanni* PCC 7110 | Complete |
| *Scytonema hofmanni* UTEX 2349 | Complete |
| *Spirulina major* PCC 6313 | Complete |
| *Spirulina subsalsa* PCC 9445 | Complete |
| *Stanieria cyanosphaera* PCC 7437 | Complete |
| *Synechococcus elongatus* PCC 6301 | Complete |
| *Synechococcus elongatus* PCC 7942 | Complete |
| *Synechococcus* sp. 1 65AY6A-5F | Complete |
| *Synechococcus* sp. BL107 | Complete |
| *Synechococcus* sp. CB0101 | Complete |
| *Synechococcus* sp. CB0205 | Complete |
| *Synechococcus* sp. CC9616 | Complete |
| *Synechococcus* sp. JA-2-3Ba(2-13) | Complete |
| *Synechococcus* sp. JA-3-3Ab | Complete |
| *Synechococcus* sp. KORDI-100 | Complete |
| *Synechococcus* sp. KORDI-49 | Complete |
| *Synechococcus* sp. KORDI-52 | Complete |
| *Synechococcus* sp. PCC 6312 | Complete |
| *Synechococcus* sp. PCC 7002 | Complete |
| *Synechococcus* sp. PCC 7002 | Complete |
| *Synechococcus* sp. PCC 7335 | Complete |
| *Synechococcus* sp. PCC 7336 | Complete |
| *Synechococcus* sp. PCC 7502 | Complete |
| *Synechococcus* sp. PE A1 60AY4Sp-7F | Complete |
| *Synechococcus* sp. PE A1 60AY6Li | Complete |
| *Synechococcus* sp. PE A1-1 60AY4M2 | Complete |
| *Synechococcus* sp. PE A1-1 63AY4M1 | Complete |
| *Synechococcus* sp. PE A1-1 65AY640 | Complete |
| *Synechococcus* sp. PE A4 65AY6A5 | Complete |
| *Synechococcus* sp. PE A6 63AY4M2 | Complete |
| *Synechococcus* sp. PE B'9 R68DH1S1RO3C-5F | Complete |
| *Synechococcus* sp. PEA 65AY6A-5F PE A | Complete |
| *Synechococcus* sp. PEA4 FF65AY629-4A PE A4 | Complete |
| *Synechococcus* sp. PEA6 M65AY6-4-5 PE A6 | Complete |
| *Synechococcus* sp. PEB5 55AY5-B PE B5 | Complete |
| *Synechococcus* sp. RCC 307 | Complete |
| *Synechococcus* sp. RS9916 | Complete |
| *Synechococcus* sp. RS9917 | Complete |
| *Synechococcus* sp. WH 8016 | Complete |
| *Synechococcus* sp. WH 8109 | Complete |
| *Synechococcus* sp. WH5701 | Complete |
| *Synechococcus* sp. WH7805 | Complete |
| *Synechocystis elongatus* PCC 7942 | Complete |
| *Synechocystis* sp. GT-S, PCC 6803 | Complete |
| *Synechocystis* sp. PCC 6803 | Complete |
| *Synechocystis* sp. PCC 6803 | Complete |
| *Synechocystis* sp. PCC 6803 | Complete |
| *Synechocystis* sp. PCC 6803 PCC-N | Complete |
| *Synechocystis* sp. PCC 6803, GT-I | Complete |
| *Synechocystis* sp. PCC 6803, PCC-P | Complete |
| *Synechocystis* sp. PCC 7509 | Complete |
| *Thermosynechococcus elongatus* BP-1 | Complete |
| *Trichodesmium erythraeum* IMS101 | Complete |
| *Xenococcus* sp. PCC 7305 | Complete |
| *Microcystis aeruginosa* PCC 7005 | Draft |
| *Nodularia spumigena* CCY9414 | Draft |
| *Prochloron didemni* P2-Fiji | Draft |
| *Prochloron didemni* P3-Solomon | Draft |
| *Prochloron didemni* P4-Papua_New_Guinea | Draft |
| *Richelia intracellularis* HM01 | Draft |
| *Arthrospira platensis* | In progress |
| *Arthrospira platensis* ABT | In progress |
| *Arthrospira platensis* AGB-AP02 | In progress |
| *Calothrix* sp. SC01 | In progress |
| *“Candidatus* Synechococcus spongiarum” LMB bulk15 | In progress |
| *Chroococcidiopsis* sp. CCMEE 29 | In progress |
| *Crocosphaera watsonii* WH0002 | In progress |
| *Cyanobacterium* sp. Missouri | In progress |
| *Cyanobacterium* sp. Texas | In progress |
| *Cyanobium* sp. Copco_Reservoir_LC18 | In progress |
| *Dermocarpa* sp. 0006 | In progress |
| *Dolichospermum circinale* ACFR02 | In progress |
| *Gloeothece* sp PCC 6909 | In progress |
| *Gloeothece* sp. PCC 6909/1 | In progress |
| *Leptolyngbya amphigranulata* | In progress |
| *Leptolyngbya* sp. ISC 25 | In progress |
| *Leptolyngbya* sp. ISC 40 | In progress |
| *Microcystis aeruginosa* FCY-26 | In progress |
| *Microcystis aeruginosa* FCY-27 | In progress |
| *Microcystis aeruginosa* FCY-28 | In progress |
| *Microcystis aeruginosa* KLA2 | In progress |
| *Microcystis aeruginosa* NIES-298 | In progress |
| *Oscillatoria* sp. ''Solar Lake' | In progress |
| *Phormidium* sp. ISC 31 | In progress |
| *Planktothrix* sp. IFCC-204 | In progress |
| *Plectonema* sp. ISC 33 | In progress |
| *Prochlorococcus* sp. UH18301 | In progress |
| *Pseudanabaena* sp. KLA6 | In progress |
| *Spirulina platensis* NIES-39 | In progress |
| *Synechococcus* sp MITS9220 | In progress |
| *Synechococcus* sp. Eum14 | In progress |
| *Synechococcus* sp. M11.1 | In progress |
| *Synechococcus* sp. M16.17 | In progress |
| *Synechococcus* sp. RCC1020 | In progress |
| *Synechococcus* sp. UW1 | In progress |
| *Synechococcus* sp. UW69 | In progress |
| *Synechococcus* sp. UW90 | In progress |
| *Synechococcus* sp. UW92 | In progress |
| *Synechocystis* sp. PCC 6803 | In progress |
| *Synechocystis* sp. PCC 6906 | In progress |
| *Tolypothrix* sp. PCC 7601 | In progress |
| *Trichodesmium thiebautii* II-3 | In progress |

**Table S2.** Secretion signal analysis of different representatives of clade 1 and 2 Clds and of Cld-like proteins. The presence or absence of a potential signal sequence was investigated using the SignalP 4.1 server (classical secretion), the TatP 1.0 server (twin arginine motif) and the Secretome 2.0 server (non-classical secretion), all provided on [www.expasy.org](http://www.expasy.org).

|  |  | **classical secretion SignalP 4.1** | **twin arginine TatP 1.0** | **non-classical secretion Secretome 2.0** |
| --- | --- | --- | --- | --- |
| **clade 2** | *Anabaena cylindrica* PCC 7122 | NO | NO | NO |
|  | *Chamaesiphon minutus* PCC 6605 | NO | NO | NO |
|  | *Cyanothece* sp. PCC 7425 | NO | NO | NO |
|  | *Gloeobacter violaceus* PCC 7421 | NO | NO | NO |
|  | *Gloeocapsa* sp. PCC 73106 | NO | NO | NO |
|  | *Leptolyngbya boryana* PCC 6306 | NO | NO* | NO |
|  | *Synechococcus* sp. PCC 6312 | NO | NO | NO |
|  | *Calothrix* sp. PCC 7103 | NO | NO | NO |
|  | *Pseudomosas aeruginosa* | NO | NO | NO |
|  | *Escherichia coli* | NO | NO | NO |
|  | *Klebsiella pneumoniae* | NO | NO | NO |
|  | *Pseudomonas stutzeri* | NO | NO | NO |
|  | *Bradyrhizobium japonicum* | NO | NO* | NO |
|  | *Nitrobacter winogradskyi* | NO | NO | NO |
| **clade 1** | *"Candidatus* Nitrospira defluvii*"* | YES | NO | NO |
|  | *Pseudomonas chloritdismutans* | YES | NO* | YES |
|  | *Alicycliphilus denitrificans* | YES | NO* | YES |
|  | *Dechloromonas hortensis* | YES | NO* | YES |
|  | *Dechloromonas aromatica* | YES | NO | YES |
|  | *Azospira orizae* | YES | NO* | YES |
|  | *Ideonella dechloratans* | YES | NO* | YES |
|  | *Magnetospirillum magnetotacticum* | YES | NO* | YES |
| **cld-like proteins** | *Listeria monocytogenes* | NO | NO | NO |
|  | *Staphylococcus aureus* | NO | NO | NO |
|  | *Geobacillus stearothermophilus* | NO | NO | NO |
|  | *Nitrospina gracilis* | NO | NO | NO |
|  | *Deinococcus radiodurans* | NO | NO | NO |
|  | *Rhodococcus jostii* | NO | NO | NO |
|  | *Thermobifida fusca* | NO | NO | NO |
|  | *"Candidatus* Nitrospira defluvii*"* 2 | NO | NO | NO |
|  | *Ferroplasma acidarmanus* | NO | NO | NO |
|  | *Staphylococcus aureus* | NO | NO | NO |

* Potential Tat signal peptide but no Tat motif was found

| **HS/LS compounds^*^** | **g_x_^eff^(HS) g_x_ (LS)** | **g_y_^eff^ (HS) g_y_ (LS)** | **g_z_^eff^ (HS) g_z_ (LS)** | **E/D** | **R (%)** | **I (%)** |
| --- | --- | --- | --- | --- | --- | --- |
| HS1 | 5.800 | 5.930 | 1.998 | 0.002 | <1 | 62 |
| HS2 | 5.650 | 6.050 | 1.998 | 0.008 | 2.5 | 28 |
| LS1 | 2.795 | 2.284 | 1.850 |  |  | 10 |

**Table S3**. EPR simulation parameters of chlorite dismutase from *Cyanothece* sp. PCC7425 (CCld) at pH 5.5 (HS – high spin, LS – low spin, E/D – rhombic to axial contribution, R – rhombicity, I – relative intensity).

^*^minimum number of high-spin and low-spin compounds used for simulation

**Fig. S1. Multiple sequence alignment of different Cld-like and Cld proteins.** Sequences were aligned using the MUSCLE algorithm. Organisms containing *cld* genes are highlighted in orange (clade 1) and green (clade 2). Protein accession numbers are taken from [http://www.ncbi.nlm.nih.gov](http://www.ncbi.nlm.nih.gov/). Organisms that carry *cld-like* genes are shown in black. Conserved residues are highlighted in yellow. The catalytically important arginine of functional Clds is marked with an orange (clade 1) or a green box (clade 2).

**Figure S2. Temperature-mediated unfolding of chlorite dismutase from *Cyanothece* sp. PCC7425 (CCld) monitored by electronic circular dichroism (ECD).** Spectra were recorded at 210 nm in the far-UV and at 411 nm in the visible region. Temperature was increased from 20 °C to 80 °C with a rate of 1 °C/min. (a,b) Thermal unfolding of CCld at pH 5.5 in the far-UV (a) and in the visible region (b). (c,d) Thermal unfolding of CCld at pH 7 in the far-UV (c) and in the visible region (d). (e,f) Thermal unfolding of CCld at pH 10 in the far-UV (e) and in the visible region (f). Insets show the corresponding van’t Hoff plots. In case of two transitions (c,d,e), full circles are used for the first transition and empty circles are used for the second one.

Figure S1, Schaffner et al.


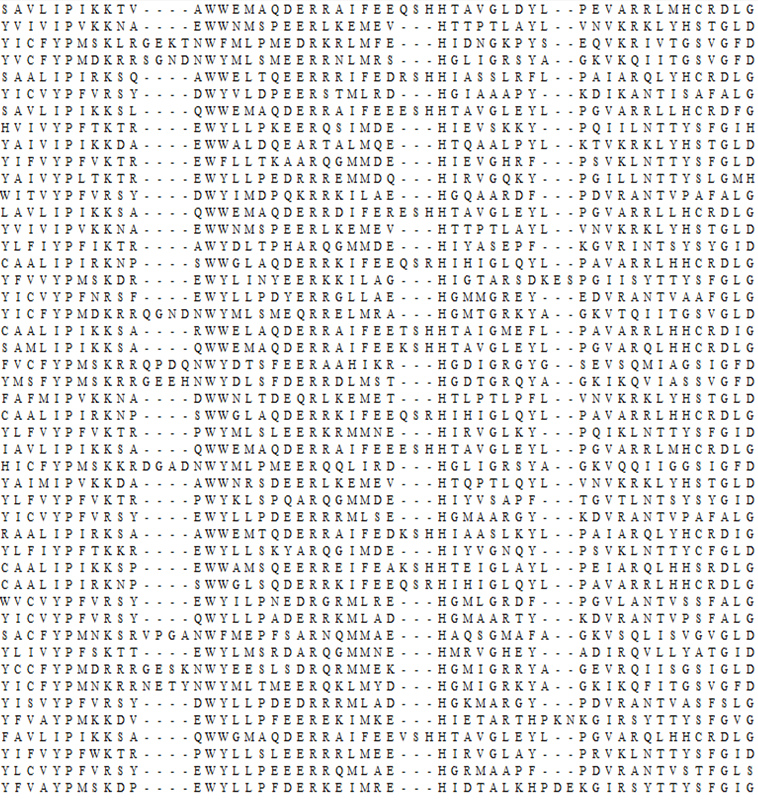


catalytic arginine (conserved in functional Clds)


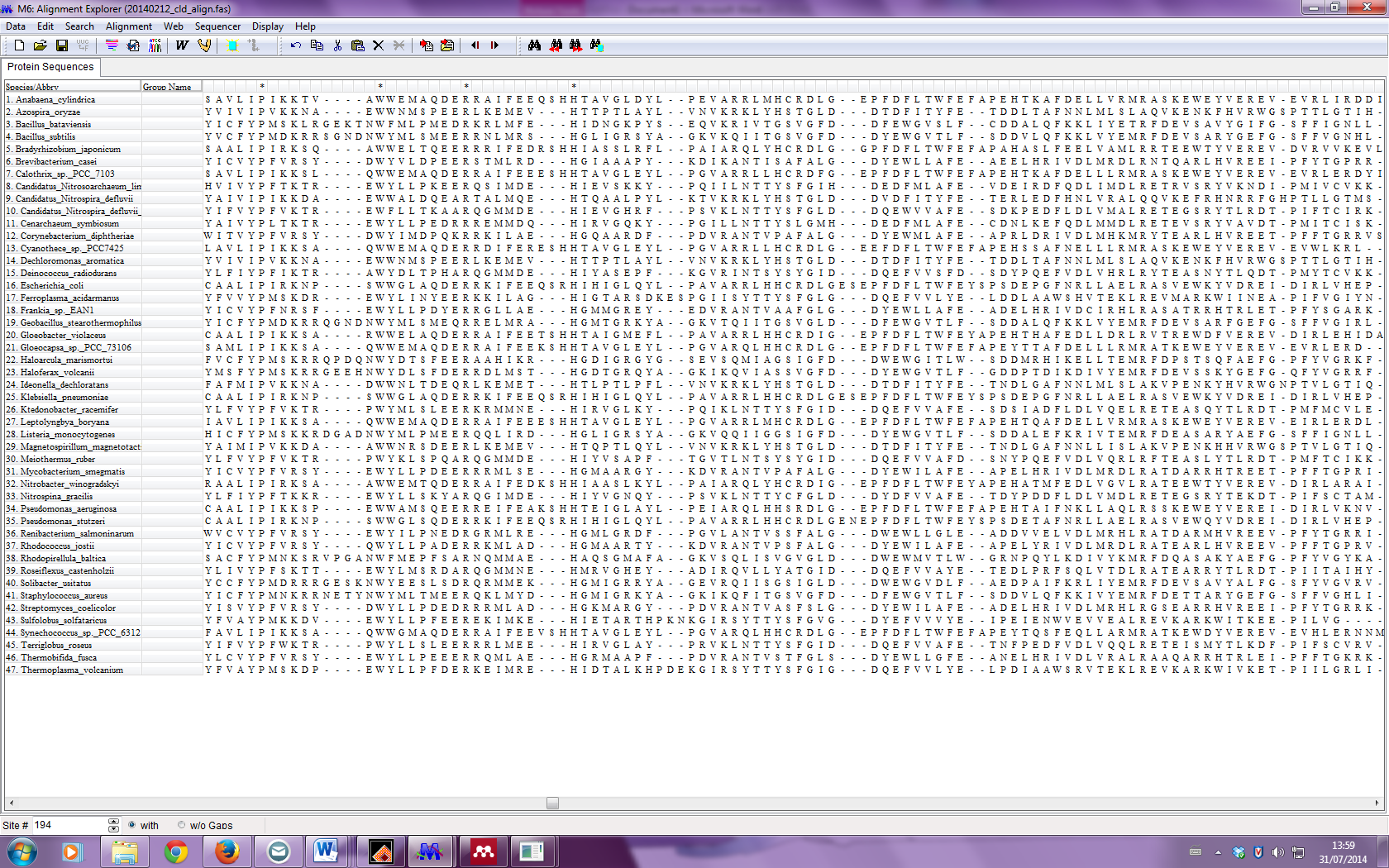


***Anabaena cylindrica*** YP_007157592.1

***Azospira oryzae*** ADN79128.1

*Solibacter usitatus* WP_011682133.1

*Bacillus bataviensis* WP_007084445.1

*Bacillus subtilis* WP_003242896.1

***Bradyrhizobium japonicum*** WP_028134486.1

*Brevibacterium casei* WP_009377031.1

***Calothrix* sp. PCC7103** WP_019497508.1

*“Candidatus* Nitrosoarchaeum limnia*”* WP_010195017.1

*“Candidatus* Nitrospira defluvii*”* WP_013249598.1

*Cenarchaeum symbiosum* WP_013482663.1

*Corynebacterium diphtheria* WP_010935038.1

***Cyanothece sp. PCC7425*** *YP_002482168.1*

***Dechloromonas aromatic*** WP_011288310.1

*Deinococcus radiodurans* NP_295204

***“Candidatus* Nitrospira defluvii*”*** WP_013247962.1

***Escherichia coli*** ESA91661.1

*Ferroplasma acidarmanus* WP_009886544.1

*Frankia* sp. EAN1 YP_001509437.1

*Geobacillus stearothermophilus* WP_015731745.1

***Gloeobacter violaceus*** NP_924112.1

***Gloeocapsa* sp. PCC73106** WP_006530645.1

*Haloarcula marismortui* AAV47812.1

*Haloferax volcanii* YP_003535903.1

***Ideonella dechloratans*** Q9F437.1

***Klebsiella pneumonia*** YP_001338852.1

*Ktedonobacter racemifer* WP_007923458.1

***Leptolyngbya boryana*** WP_017285540.1

*Listeria monocytogenes* WP_003730371.1

*Meiothermus ruber* YP_003507186.1

*Mycobacterium smegmatis* WP_003894166.1

***Nitrobacter winogradskyi*** YP_319047.1

*Nitrospina gracilis* WP_005010869.1

***Pseudomonas aeruginosa*** CAH04648.1

***Pseudomonas stutzeri*** YP_001173821.1

*Renibacterium salmoninarum* WP_012244645.1

*Rhodococcus jostii* YP_707279.

*Rhodospirellula baltica* CAD76620.1

*Roseiflexus castenholzii* YP_001432133.1

*Staphylococcus aureus* NP_645359.1

*Streptomyces coelicolor* WP_003972879.1

WP_003972879.1

*Sulfolobus solfataricus* NP_343554.1

***Synechococcous sp. PCC6312*** YP_007060490.1

*Terriglobus roseus* WP_014786045.1

*Thermobifida fusca* WP_011292320.1

*Thermoplasma volcanium* WP_010917049.1

proximal heme ligand

part of proximal H-bonding network

(a) (b)

(c) (d)

(e) (f)

Figure S2, Schaffner et al.
